# An Alternative Lifetime Model for White Light Emitting Diodes under Thermal–Electrical Stresses

**DOI:** 10.3390/ma11050817

**Published:** 2018-05-16

**Authors:** Xi Yang, Bo Sun, Zili Wang, Cheng Qian, Yi Ren, Dezhen Yang, Qiang Feng

**Affiliations:** School of Reliability and Systems Engineering, Beihang University, Beijing 100191, China; bryantyx@buaa.edu.cn (X.Y.); sunbo@buaa.edu.cn (B.S.); wzl@buaa.edu.cn (Z.W.); cqian@sklssl.org (C.Q.); renyi@buaa.edu.cn (Y.R.); dezhenyang@buaa.edu.cn (D.Y.)

**Keywords:** lifetime prediction, light emitting diodes, thermal stress, electrical stress, accelerated degradation test

## Abstract

The lifetime prediction using accelerated degradation test (ADT) method has become a main issue for white light emitting diodes applications. This paper proposes a novel lifetime model for light emitting diodes (LEDs) under thermal and electrical stresses, where the junction temperature and driving current are deemed the input parameters for lifetime prediction. The features of LEDs’ lifetime and the law of lumen depreciation under dual stresses are combined to build the lifetime model. The adoption of thermal and electrical stresses overcomes the limitation of single stress, and junction temperature in accelerated degradation test as thermal stress is more reliable than ambient temperature in conventional ADT. Furthermore, verifying applications and cases studies are discussed to prove the practicability and generality of the proposed lifetime model. In addition, the lifetime model reveals that electrical stress is equally significant to the thermal stress in the degradation of LEDs, and therefore should not be ignored in the investigation on lumen decay of LEDs products.

## 1. Introduction

White LED is proverbially acknowledged as the most promising energy saving solution for lighting applications due to its high efficiency and long lifetime [1]. The inherent high reliability and long lifetime require the white LED to have a lower luminous flux degradation and smaller color shift during operational period [2,3]. Prognostics and reliability studies on LEDs benefit both manufactures and consumers by improving the accuracy of lifetime prediction, optimizing LEDs design, and shortening qualification test times [4], which have attracted global attentions. A previous paper [5] summarized the recent research and development in the field and discussed the pros and cons of various prognostic techniques.

The conventional reliability estimation test methods cost a test time of at least 6000 h, which is apparently too long for LEDs manufactures [6,7]. Therefore, there is an urgent need for fast and effective test methods with time and cost considerations [5]. Some essential and superior methods were developed to help people have a deep understanding on the nature of reliability of LEDs [8,9], such as the photoelectrothermal (PET) theory for LED systems [10,11,12], the degradation of silicone in white LEDs during operation [13], the degradation mechanism of silicone glues [14] and the coupling effects of both LED and driver’s degradations [15].

As an effective substitution to reduce the test duration of white LEDs and relevant products, the constant stress accelerated degradation test (CSADT) has been recognized to be efficient in minimizing the sample size and shorten test time [16,17,18], and therefore are employed under overstress conditions for LED products [19,20]. For instance, Qian et al. developed a CSADT method to reduce the reliability test period from 6000 h to 2000 h for LED luminaires and lamps [21]. Via a boundary curve theory, they proved that the qualification results obtained from the 6000 h test data under 25 °C and 1500 h test data under 55 °C are comparable to each other for a majority of LED lighting products. However, these studies based on CSADT method focused only on single stress and did not build up relationship between lifetime and accelerated stresses.

Through the historical investigations on the degradation characteristics of LEDs, the accelerated loadings could be high temperature [22], high moisture [23], high driving current [24], a combination of temperature and moisture [25,26] or a combination of temperature with current [27]. Edirisinghe et al. used a junction temperature based Arrhenius model to determine the lifetime of 1-W HBLEDs (high-bright light-emitting diodes) [28]. An accelerated aging test for high-power LEDs under different high-temperature stresses without input current was conducted in [29], which shows that a sufficiently high-temperature stress effectively shortens the unstable period of the LED chip. Sau Koh et al. presented an accelerated testing by taking the ambient temperature of 55 °C as the accelerated stress, combining the exponential decay model and Arrhenius equation by using a two-stage acceleration theory [30]. Thermal stress is regarded as having the largest influence on the degradation of LED, and the mentioned papers often set the ambient temperature as the accelerated stress, which, in fact, should be junction temperature. Furthermore, these studies investigated the acceleration of single stress but ignored the coupled influences of the current stresses on each other.

In addition, the operating conditions of multiple-coupled stresses ADT on LEDs are much more sophisticated than that of single stress. Wang et al. conducted a thermal–electrical stressed accelerated degradation test on LED-based light bars. In their study, the effect of current was transferred into junction temperature [31]. An accelerated life test method with temperature and currents is reported in [32], i.e., the lifetime of the LEDs was extrapolated by first a temperature stress acceleration and then a current stress acceleration. Besides, three-stage degradation behavior of GaN-based LEDs under a thermal–electrical dual stress is demonstrated in [33]. These studies revealed the effects of thermal–electrical dual stress on LED product. However, there is no specific model to describe the connection between lifetime and thermal–electrical dual stress.

The successful applications of CSADT models reveal that acceleration method is quite effective in shorting the duration of testing to obtain data for LED lifetime estimation. The research focus concentrates on solving the dilemma between accuracy and universality and enhancing the efficiency of the ADT method. On the other hand, the current CSADT methods are based on the empirical models, of which the parameters were often obtained by curve fitting from experimental data [34]. These models conclude the relationship between degradation and operation time, ignoring the loading conditions in the emission profiles [35].

To deepen the understanding on the relationship between lifetimes of LEDs and their operating conditions, the degradation rate of LED under constant thermal and electrical stresses is investigated in this paper. Then, this paper proposes a lifetime prediction model under dual stresses, which reveals the physical principle of degradation and loading conditions in LEDs. Furthermore, the linear accumulated damage model was combined with the lifetime model to predict lifetime under emission profiles of LEDs. With the lifetime model concerning dual stresses, the lifetime prediction based on junction temperature and driving current becomes more reliable and correct.

The remainder of this paper is organized as follows: Section 2 establishes the lifetime model under thermal and electrical stresses, followed by the solution and analysis on model parameters in Section 3. The applications and case studies are discussed in Section 4. The concluding remarks are drawn in Section 5.

## 2. The Lifetime Model for LEDs under Thermal and Electrical Stresses

It is reported in TM-21-11 that the gradual luminous flux degradation follows an exponential decaying function [7]:

(1) $$\Phi =\beta e^{-\alpha t},$$

where Φ is the lumen maintenance, β is the pre-factor, and α denotes the decay parameter. For LEDs products, the failure criterion, denoted as *L*_p_, where *p* equals 50 or 70 based on different applications, is defined as the time when lumen maintenance degrades to *p%*, as given by:

(2) $$L_{p}=\ln(\beta /p)/\alpha ,$$

When applied by thermal and electrical stresses, the degradation rate of the LEDs can be calculated using Eyring model:

(3) $$\alpha =AI^{n}\exp\left(-\frac{E_{a}}{kT_{j}}\right),$$

where *T*_j_ is the absolute temperature in Kelvin; *I* represents the driving current in LEDs products; $E_{a}$ *E*_a_ and *k* denote the activation energy and Boltzmann constant, respectively; and *n* and *A* are the model constants.

By substituting Equation (3) into Equation (2), the lifetime model under thermal and electrical stress can be established as:

(4) $$L_{p}=\ln(\beta /p)/A\cdot I^{-n}\cdot \exp\left(\frac{E_{a}}{kT_{j}}\right),$$

Next, by using Λ to replace ln(β/p)/A, where τ denotes $L_{p}$, Equation (4) can be simplified as:

(5) $$\tau =\Lambda I^{-n}\cdot \exp\left(\frac{E_{a}}{kT_{j}}\right),$$

## 3. Solution and Analysis of the Lifetime Model

### 3.1. Model Solving

Equation (6) can be rearranged as Equation (7) by taking natural logarithms on both sides of the equation.

(6) $$\ln\tau =\ln\Lambda -n\ln I+\frac{E_{a}}{kT_{j}},$$

Letting z=lnτ, x=lnI, $y=\frac{1}{T_{j}}$, $\gamma_{1}=\ln\Lambda$, $\gamma_{2}=-n$, and $\gamma_{3}=\frac{E_{a}}{k}$, the model in Equation (6) can be transformed into the following formula:

(7) $$z=\gamma_{1}+\gamma_{2}x+\gamma_{3}y,$$

Therefore, the estimation of parameters in the model in Equation (7) can be translated into a multiple linear regression.

In the process of solving function, at least three sets of data are needed. Three sets of $\left(x_{i},y_{i},z_{i}\right)$ can be used to estimate $\Gamma =(\gamma_{1},\gamma_{2},\gamma_{3})$. The algorithm can be expressed in matrix form:

(8) $$Z=\Gamma •{\left(1,X,Y\right)}^{T},$$

where $Z=(z_{1},z_{2},z_{3})$, $X=(x_{1},x_{2},x_{3})$, and $Y=(y_{1},y_{2},y_{3})$.

After accelerated degradation testing for LED products, the data of thermal stress, electrical stress and lumen degradation can be obtained. Furthermore, the lifetime under each stress level can be extrapolated using the degradation trajectories of the LED products. These data can be transformed into parameters in the model algorithm to solve $\gamma_{1}$, $\gamma_{2}$ and $\gamma_{3}$.

Furthermore, the lifetime estimation model under thermal–electrical stresses can be expressed in Equation (9).

(9) $$\tau =I^{\gamma_{2}}\exp\left(\frac{\gamma_{3}}{T_{j}}+\gamma_{1}\right),$$

As has been reported in the literature [31], Wang et al. designed five sets of thermal and electrical stress levels to conduct accelerated degradation testing for LED-based light bars, where the junction temperature and driving current are designed as accelerated stresses. The first three sets of stresses are selected to solve the lifetime model proposed in this paper, while the remaining two sets of data are employed to verify the model.

**Figure 1:**
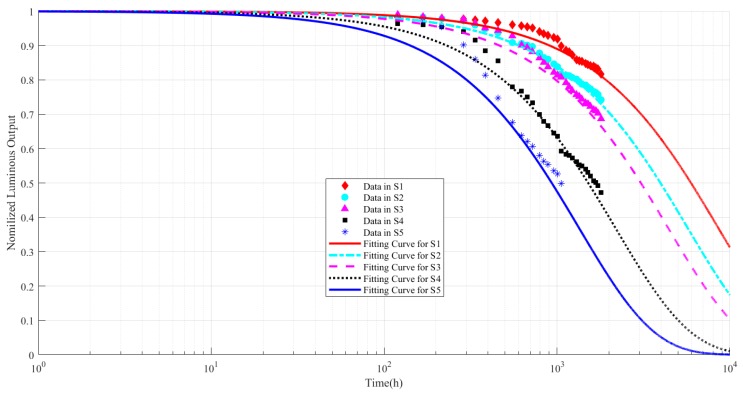
Degradation data [31] and trajectories under different stress levels.

The driving currents, ambient temperatures and junction temperatures in each stress level are listed in Table 1. Meanwhile, Figure 1 demonstrates the average normalized light outputs under different stress levels and the fitting degradation trajectories.

As shown in Figure 1, there are apparent discrepancies between the fitting curves and data in S3, S4 and S5 for the early time, while, for longer time, the experimental data fits well with the curves. This phenomenon may be explained with the assumption that activation energy shift slows down the luminous flux decay, and for longer time the activation energy turns back to its initial state.

The expected lifetimes in different stress levels can be obtained using Equations (1) and (2), which are listed in Table 2. By taking $\left(x_{i},y_{i},z_{i}\right)$ in Equation (8), the parameters are extracted as $\gamma_{1}$= −2.5774, $\gamma_{2}$= −0.1699 and $\gamma_{3}$= 4197.9.

Therefore, the lifetime prediction model for the particular test samples in Wang’s study under thermal and electrical stresses is solved as:

(10) $$\tau =I^{-0.1699}\exp\left(\frac{4197.9}{T_{j}}-2.5774\right),$$

The quantitative effects of driving current and junction temperature on lifetime can be expressed in Equation (10), with which the lifetime can be predicted under the given thermal–electrical coupling conditions. In this paper, the expected lifetime comes from the experimental data while the predicted lifetime is obtained using proposed model. When the junction temperature and driving current in S4 and S5 are used in Equation (10), the lifetimes are predicted as 3038.0 h and 1965.2 h, respectively. There is an obvious deviation between the predicted lifetime and the expected lifetime, i.e., 91.9% for S4 and 92.1% for S5.

Compared with S3, S4 has higher junction temperature and lower driving current, but the expected lifetime in S4 is much smaller than that in S3, thus it can be deduced that the degradation mechanism were changed by the high junction temperature which causes dramatic lifetime drop in S4. Therefore, the lifetime model should be modified to characterize the mechanism variation in the experiment. The correction factor $\varepsilon (T_{j},I)$ is introduced into the lifetime model:

(11) $$\tau =I^{-0.1699}\exp\left(\frac{4197.9}{T_{j}}-2.5774+\varepsilon (T_{j},I)\right),$$

$\varepsilon (T_{j},I)$ is related with the junction temperature and driven current. Besides, when the stress level is smaller than stress level that changes the degradation mechanism, $\varepsilon (T_{j},I)=0$. When strong stress level is exerted to the LEDs products, $\varepsilon (T_{j},I)$ can be estimated with a new set of data. Due to insufficient experimental data, the detailed degradation mechanism shift cannot be further determined in this paper. The reasons may lie in the effect of high driving current on LED chips, or high temperature on packaging materials, or the coupling effects of both factors.

With the data in S4, the correction factor is estimated as $\varepsilon (T_{j},I)=\;-0.5778$, the correction factor can be explained as a constant to characterize the changed degradation mechanism. Consequently, the lifetime is predicted with the modified lifetime model in Equation (11) as 1077.8 h with parameters in S5, which is quite close to the expected lifetime with an error of (1077.8 − 1023.2)/1023.2 = 5.34%.

### 3.2. Compare with Wang’s Model

**Figure 2:**
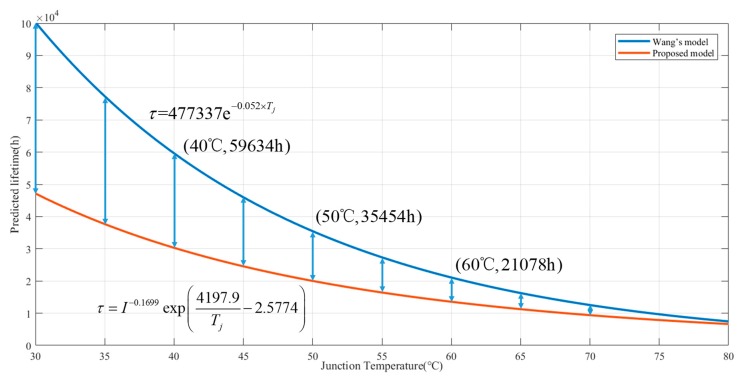
Comparison of Wang’s model [31] and the model proposed in this paper.

In addition, compared with the lifetime model in Wang’s study, where the lifetime was given by $\tau =477337e^{-0.052\times T_{j}}$, the lifetime predicted by the dual-stress model is lower than that of single-stress model under the same emission profile, as shown in Figure 2. The phenomenon is due to the ignorance of the driving current in degenerating the lifetime of LEDs in Wang’s study. Therefore, the driving current should be considered when modeling with the thermal and electrical stresses ADT data. In addition, the lifetime gap of the two models becomes smaller as the junction temperature increases; this tendency is due to the dominant status in the high temperature area of junction temperature.

### 3.3. Effects of Junction Temperature and Driving Current on Lifetime

**Figure 3:**
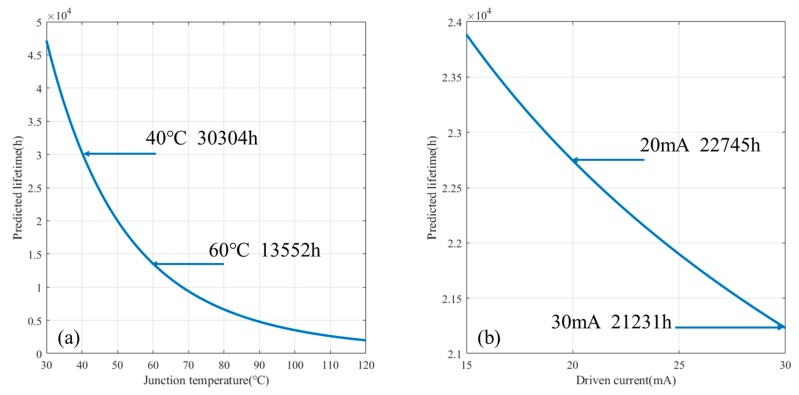
Expected lifetime decays with: junction temperature (**a**); and driving current (**b**).

On the one hand, when LEDs work under a constant driving current, Equation (10) follows the form τ = γe1Tj, which reveals that lifetime decays with junction temperature in an exponential form as shown in Figure 3a. Figure 3a also exhibits that the sensitivity of lifetime loss is much more significant at the low temperature are than that at the high temperature area. Therefore, the attempt to lower junction temperature is of practical significance in maintaining longer lifetime.

On the other hand, when LEDs works under a constant junction temperature, Equation (10) follows the form $\tau \;=\;\beta I^{\gamma_{2}}$, in which $\gamma_{2}$ is a negative constant and β is positive. This indicates that lifetime decays with the driving current in a power form, as shown in Figure 3b. However, dissimilar to the effect of junction temperature, curve of lifetime degradation in Figure 3b is nearly linear because the order of the power function is too small. Therefore, the lifetime of LEDs can be considerably prolonged by reducing the driving current within a reasonable range.

The lifetime model and curves in Figure 3 reveal that lifetime decreases monotonically with the increasing junction temperature and driving current, but the descent rates of the curves are different. Although the driving current has a power law effect, whereas the junction temperature has an exponential effect, the expected lifetime of LEDs is much more sensitive to the junction temperature than driving current. For instance, the expected lifetime will increase from 13,552 h to 30,304 h as junction temperature drops from 60 °C to 40 °C (a decrease of 33.3%), while a decrease of 33.3% in driving current (from 30 mA to 20 mA) leads a lifetime increase from 21,231 h to 22,745 h.

The result suggests that it is more feasible to maintain a longer lifetime by reducing the junction temperature. In addition, there are two ways to reduce junction temperature: better cooling condition and lower driving current. Because a lower driving current in LEDs leads longer lifetime, it is more practical to reduce driving current when the cooling condition cannot be optimized for the enhance of lifetime.

## 4. Applications and Case Study of the Lifetime Model

Generally speaking, an accurate and robust estimation on the expected lifetime of LED products is vitally important when using them in practical applications, especially under harsh conditions. However, due to complications from both internal and external environmental stresses, it is not easy to perform a comprehensive prediction on the expected lifetimes. This section provides a solution based on Equation (10) on the expected lifetime prediction for LEDs under a complicated emission profile with any given combinations between varying temperatures and driving currents.

For a given operational condition of ambient temperature and driving current, the corresponding lifetime can be predicted using the lifetime model. However, in the outdoor application of LEDs, the ambient temperature and loading conditions often change, causing variation of junction temperature and driving current. Based on assumption of linear accumulated damage model [36], the consumed lumen lifetime can be predicted as:

(12) $$CL=\sum_{i=1}^{n}\frac{t_{i}}{\tau_{i}},$$

where n is the number of stress levels, $t_{i}$ is the accumulated duration at stress ${\left(T_{j},I\right)}_{i}$, and $\tau_{i}$ is the corresponding lifetime. Therefore, the lumen lifetime under varying ambient and loading conditions is the time when *CL* reaches 1. Assuming that the duration of ambient conditions is periodic, the total lifetime can be calculated by $\tau =\frac{1}{CL}\sum_{i=1}^{n}t_{i}$.

**Figure 4:**
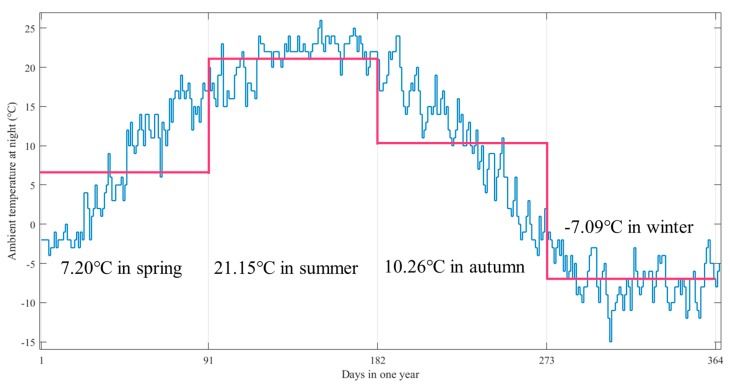
The average ambient temperature at night every day in Beijing.

Unlike the indoor application, the outdoor application such as street lamp endures variable ambient temperature at different seasons in one year. The LED lamps works at rated current from 19:00 p.m. to the next day 5:00 a.m., lasting 10 h per day, while the ambient temperature varies with seasons. Take Beijing as an example: the average ambient temperatures at night from spring to winter are 7.20 °C, 21.15 °C, 10.26 °C and −7.09 °C respectively, as demonstrated in Figure 4. For the sake of facilitating calculation, the assumption that a quarter contains 91 days and exclusion of current fluctuation is reasonable.

Under this circumstance, the number of stress level n is 4, and the accumulated duration at each stress level $t_{i}$ equals 910 h. Based on the proposed model, the corresponding predicted lumen lifetimes at each stress level are listed in Table 3.

Based on Formula (12), the consumed life can be calculated as CL=t1τ1+t2τ2+t3τ3+t4τ4=0.1184, thus the prediction of total lumen lifetime under given emission profile is τ=4tCL=30,735h.

## 5. Conclusions

In this paper, the expected lifetime related to lumen degradation of LEDs under both thermal and electrical stresses are investigated and predicted using the dual-stress lifetime model. With the experimental data in [31], the lifetime model was established and validated, which proved the availability and effectiveness of the model. The experimental data also exhibit that the degradation mechanism was changed by the high level of acceleration stresses, but the critical value of stress level was not determined due to the lack of necessary data. A correction factor was introduced to enhance the accuracy of lifetime prediction under high stress level, with which the relative error drops from 92.1% to 5.34%. When the stress level is lower than the critical value, the correction factor equals zero, or, other cases, more data are required to determine the new critical stress level.

In addition, the predicted lifetime is smaller with the dual-stress lifetime model than that with Wang’s model due to the coupling effect of driving current on both lifetime and junction temperature. Therefore, it is revealed that driving current is another crucial factor when predicting lifetime and cannot be ignored in modeling with thermal and electrical stresses ADT data. 

The comparison between the effects of junction temperature and driving current on lifetime indicates that the lifetime of LEDs is more sensitive to the thermal stress, i.e., junction temperature, while electrical stress also causes depreciation in lifetime. In the discussion, the lifetime decays with junction temperature in an exponential form, a slight fluctuation in junction temperature will cause great drop in lifetime. Therefore, more efforts should be paid on reducing the junction temperature of LEDs.

Furthermore, a method that combines linear accumulated damage theory with the dual-stress lifetime model is proposed to predict lifetime of LEDs product under variable stresses. The case study makes it unequivocal that the lifetime model can be successfully applied to the circumstances of constant and variable stresses.

However, this paper considers only the luminous depreciation in LED for lifetime prediction, and the model cannot characterize other failure models such as color shift and catastrophic failure. Besides, the statistic properties of life data from accelerated degradation testing were not considered during the model solving. The lifetime prediction model based on color shift and exploration for advanced statistical methods will be conducted in the prospective work.

## Figures and Tables

**Table 1:** Description of stress level conditions.

Stress Level	Experimental Conditions (Driving Current, Ambient Temperature)	Quantity of Sample	Junction Temperature *T*_j_ ( °C)
S1	(20 mA, 60 °C)	15	82.4
S2	(30 mA, 60 °C)	15	93.6
S3	(25 mA, 72.5 °C)	15	100.5
S4	(20 mA, 85 °C)	15	107.4
S5	(30 mA, 85 °C)	15	118.6

**Table 2:** Independent variables for model solution.

Item	Stress Level				
	S1	S2	S3	S4	S5
Expected lifetime (*L_50_*)	6126.8 h	3987.7 h	3329.5 h	1582.9 h	1023.2 h
Driving current (I)	20 mA	30 mA	25 mA	20 mA	30 mA
Junction temperature ($T_{j}$)	355.33 K	366.75 K	373.65 K	380.55 K	391.75
Predicted lifetime	–	–	–	3038.0 h	1965.2 h

**Table 3:** Predicted lumen lifetimes under various emission profiles.

No.	Season	Accumulated Duration	Stress Level (*T*_a_, *I*)	Predicted Lifetime (τ)
1	Spring	910 h	(7.20 °C, 20 mA)	34,201 h
2	Summer	910 h	(21.15 °C, 20 mA)	19,113 h
3	Autumn	910 h	(10.26 °C, 20 mA)	29,969 h
4	Winter	910 h	(−7.09 °C, 20 mA)	65,702 h

## Nomenclature

Φ	Lumen maintenance
β	Pre-factor of LED’s depreciation
α	Degradation rate of LEDs
$L_{p}$	Failure criterion of LEDs
$T_{j}$	Junction temperature in LEDs
I	Driving current
$E_{a}$	LED’s activation energy
k	Boltzmann constant
n	Model constant
A	Model constant
